# The evaluation of "Safe Motherhood" program on maternal care utilization in rural western China: a difference in difference approach

**DOI:** 10.1186/1471-2458-10-566

**Published:** 2010-09-22

**Authors:** Xiaoning Liu, Hong Yan, Duolao Wang

**Affiliations:** 1 Department of Epidemiology and Health Statistics, School of Public Health, Xi'an Jiaotong University College of Medicine, Xi'an, Shaanxi 710061, China; 2 Institution of Epidemiology and Health Statistics, School of Public Health, Lanzhou University, Lanzhou, Gansu 730000, China; 3 Department of Epidemiology and Population Health, London School of Hygiene and Tropical Medicine, London WC1E 7HT, UK

## Abstract

**Background:**

Maternal care is an important strategy for protection and promotion of maternal and children's health by reducing maternal mortality and improving the quality of birth. However, the status of maternal care is quite weak in the less developed rural areas in western China. It is found that the maternal mortality rates in some western areas of China were 5.8 times higher than those of their eastern costal counterparts. In order to reduce the maternal mortality rates and to improve maternal care in western rural areas of China, the Chinese Ministry of Health (MOH) and the United Nations Children's Fund (UNICEF) sponsored a program named "Safe Motherhood" in ten western provinces of China from 2001 through 2005. This study mainly aims to evaluate the effects of "Safe Motherhood" program on maternal care utilization.

**Methods:**

32 counties were included in both surveys conducted in 2001 and 2005, respectively. Ten counties of which implemented comprehensive community-based intervention were used as intervention groups, while 22 counties were used as control groups. Stratified 3-stage probability-proportion-to-size sampling method was used to select participating women. Two cross-sectional surveys were conducted with questionnaires about the prenatal care utilization in 2001 and 2005, respectively. Difference in difference estimation was used to assess the effect of intervention on the maternal care utilization while controlling for socio-economic characteristics of women.

**Results:**

After the intervention, the proportion of pregnant women who had their first prenatal visit in the first trimester was increased from 38.9% to 76.1%. The proportion of prenatal visits increased from 82.6% to 98.3%. The proportion of women mobilized to deliver in hospitals increased from 62.7% to 94.5%. Hospital delivery was improved greatly from 31.1% to 87.3%. The maternal mortality rate was lowered by 34.9% from 91.76 to 59.74 per 100,000 live births. The community-based intervention had increased prenatal visits rate by 5.2%, first prenatal visit in first trimester rate by 12.0% and hospital delivery rate by 22.5%, respectively. No effect was found on rate of women being mobilized to hospital delivery compared with that of the control group.

**Conclusion:**

The intervention program seemed to have improved the prenatal care utilization in rural western China.

## Background

There is a strong link between low use of maternal health services for delivery and high maternal mortality [1]. Globally, about 60 million women give births outside health facilities, mainly at home, and 52 million births occur without a skilled birth attendant every year [2]. Moreover, about half a million women--many of them living in developing countries--died from maternal causes each year [3].

On the other hand, socioeconomic and geographic inequalities have also contributed to high maternal mortality. That might be due to the large differences in the use of maternal care especially in rural areas and poorer regions. In 2001, the hospital delivery rate was 76.0% for the whole nation in China, while it was 87% for the urban population, 69.0% in rural areas and 48.8% in western rural areas. In the meantime, the maternal mortality rate (MMR) was 50.2 per 100,000 live births for the whole nation, while it was 33.1 per 100,000 live births in urban population and 61.9 per 100,000 live births in rural areas [4]. The ratio of urban to rural maternal mortality is nearly 1: 1.87. MMR has remained above 100 per 100,000 live births in Tibet, Qinghai, Xinjiang, Guizhou and Gansu provinces [5]. It has been shown that in China, 80% of maternal deaths were farmers, 58.5% of whom delivered at home and 35.5% died at home [6]. Most obstetric complications occur at the time of delivery and can not be predicted. Previous studies have also confirmed that lack of prenatal health care and without the benefit of any skilled birth attendance during delivery were the main causes of maternal death. Obstetric hemorrhage is the leading cause of maternal death, accounting for more than one fifth of all causalities [4]. The differences in maternal health are more obvious. It was an urgent problem of lagged maternal health care in rural areas of China.

Chinese Ministry of Health (MOH) and the United Nations Children's Fund (UNICEF) carried out a program named "Safe Motherhood" in rural western China from 2001 to 2005. 46 counties in 10 western provinces were included in this program, 10 counties of which implemented comprehensive community-based intervention. The main message that this program was trying to convey was that hospital delivery would be the safest way for delivery and an efficient way to reduce maternal mortality. Therefore, the purpose of this study is to evaluate the effects of this program on utilization of prenatal care.

## Methods

### Subjects and design

The program was implemented in ten provinces of rural western China. At the beginning of the intervention in 2001, 46 counties of 10 western provinces were chosen. Then in 2005, 45 counties were chosen to evaluate the effects of this program. The counties were not selected randomly but determined by MOH and UNICEF. Two cross-sectional surveys were conducted at the beginning (pre-intervention) and end (post-intervention) of the intervention, respectively. 32 counties were included in both surveys in 2001 and 2005. This study is based on the family questionnaire survey data in these 32 counties from 2001 through 2005. Among these 32 counties, ten counties were exposed to the intervention and 22 counties were used as controls.

Considering the hierarchical structure of Chinese administrative districts and the imbalanced population distributions among the different provinces, a stratified 3-stage probability-proportion-to-size sampling method was employed for sampling in this study. Five townships were selected randomly in each county; four villages were selected randomly in each township, and then a woman with children who were less than three years old was selected from 16 random families in each village. All women were interviewed face-to-face by trained professional interviewers from the Xi'an Jiaotong University College of Medicine. A questionnaire was used to collect information on family socio-demographic characteristics and prenatal health care. All participates in this project gave their written informed consent after reading through the consent forms. Consent was obtained before administration of the survey questionnaire. The study was reviewed and approved by the Ethics Committee at the Xi'an Jiaotong University College of Medicine. The number of live births and maternal death per year were obtained from health bureau of each target county, where data were recorded.

The objectives of the program were to improve the prenatal health care and hospital delivery in township hospital or above level health facility, and reduce the MMR. The intervention measures consisted of:

1. Establishing the three-level "county-township-village" rural health care network in target counties, by which all national health policies are implemented;

2. Restricting on hospital delivery costs or providing of appropriate subsidies for poor pregnant women in target counties;

3. Training the heads of target county and health bureau to improve their abilities of project management;

4. Establishing the emergency obstetric centers in target counties, where the obstetric medical staff of township hospital will be trained. In this way, obstetric technical guidance for township hospital will be provided, and the severely ill patients who can't cure in township hospital will get treatments.

5. Strengthening the maternal health-service quality in township hospitals, improving and providing quality basic emergency obstetric care in township hospitals;

6. Changing the functions of village doctors, so that they can replace the midwife. In that way, they could give some health suggestions to pregnant woman face to face and encourage them to deliver in hospital.

### Statistical analysis

Data were entered into EPI Info Version 6.0 (CDC, Atlanta, GA, USA) and analyzed with SPSS Version 13.0 (Statistical Package for Social Science, Inc, Chicago, IL, USA) and STATA version 9.2 (Stata/SE 9.2 Stata Corporation, College Station, TX, USA). The main outcome variables were as follows: (1) whether a pregnant woman had a prenatal visit; (2) whether a pregnant woman had a prenatal visit during the first trimester; (3) whether a pregnant woman was mobilized to hospital delivery; (4) whether a pregnant woman gave birth in a township hospital or higher level hospital. These outcome variables are binary outcomes and were summarized by percentage of women who had an outcome, and those differences in proportions were compared between different groups using the Chi-squared test. The differences in MMR were compared between different groups using two-sample test of Poisson distribution. Results were considered statistically significant when the two-sided *P *value was less than 0.05.

Difference in difference (D-in-D) estimation was used to assess the effects of intervention on the outcome variables while controlling for socio-economic characteristics of women in the framework of linear regression model. We define *i *= 1 for the intervention group and *i *= 0 for the control group, and *t *= 1 as year 2005 and *t *= 0 as year 2001. Let *μ_it _*be the mean of an outcome variable in group *i *at time *t*. The difference in means between intervention and control group in 2005 (*μ*_11 _- *μ*_01_) is the unadjusted estimate of the intervention effect. Adjusting for the baseline difference, the adjusted estimate of intervention effect is (*μ*_11 _- *μ*_10_) - (*μ*_01 _- *μ*_00_), i.e. the D-in-D estimate of intervention effect. The D-in-D estimator is actually the coefficient of the interaction term between intervention and time in a linear regression model with intervention, time, and their interaction as covariates. For a binary outcome variable, the D-in-D estimate is the difference in 2005 in changes from 2001 in proportion of women having an event. In addition, the following covariates were included in the regression model: ethnicity, average annual income of village per year, women's age, parity, education, and the distance between village and township. These variables were selected based on the previous studies but constrained by the variables collected in the survey.

## Results

The two surveys were conducted in the same season during July-August 2001 and 2005, respectively. It was expected that five townships in each county and four villages in each township would be surveyed, but some of the selected townships had no hospital and some of the selected villages had no health clinic at all. Table 1 shows the sample information on surveyed counties, townships, villages and women.

**Table 1:** The number of surveyed counties, townships, villages and women in 2001 and 2005

	2001		2005	
	
	Intervention	Control	Intervention	Control
Counties	10	22	10	22
Townships	48	105	50	108
Villages	167	370	189	432
Women	3253	7013	3043	6737

In the baseline survey, the percentage of women who had a prenatal visit was 82.6% in the intervention group and 88.7% in the control group. After the intervention, the percentage was increased to 98.3% but there was no significant difference between the intervention and the control group (98.4%). On the other hand, the percentage of women who had a prenatal visit during the first trimester was 38.9% in the intervention group but less than that of 43.7% in the control group in 2001. In 2005, this percentage was increased to 76.1% in the intervention group and more than 67.6% in the control group (*P *< 0.001). Among those women, 17.8% had a prenatal visit at home or village clinic, 39.6% in township hospital and 34.6% in county health facility in 2001. After the intervention, the proportion of place where women had their prenatal visits had no difference compared with that in the control group (*P *= 0.513). However, the proportion of women had a prenatal visit at township hospital or county health facility increased by 24.9% in the intervention group, only 14.7% in the control group. In 2001, more than half of the pregnant women (67.6%) gave birth at home or village clinic in the intervention group, which was significantly higher than the control group (40.3%) (*P *< 0.001). Then in 2005, nearly half of pregnant women in the intervention group (47.5%) chose to deliver in township hospital and 39.8% of them chose to deliver in county health facility. It is obvious from the results that the percentage of pregnant women who gave birth at home or village clinic was sharply reduced to 11.5%, which was slightly higher than the control group (8.7%) (*P *< 0.001). About 94.5% of the women who were mobilized to have a hospital delivery after intervention were higher than that in the control group (88.9%) (*P *< 0.001). The MMR was lowered by 34.9% from 91.8 to 59.7 per 100,000 live births in the intervention group, while the MMR was reduced by 14.0% from 90.2 to 77.7 per 100,000 live births in the control group. (Table 2)

**Table 2:** The utilization of maternal health care and maternal mortality rate in 2001 and 2005

Outcomes	2001			2005			Change	
	
	Intervention	Control	*P*-value	Intervention	Control	*P*-value	Intervention	Control
**Had a prenatal visit (%,n)**	82.6 (2687)	88.7 (6220)	0.001	98.3 (2991)	98.4 (6629)	0.703	19.0	10.9
**First prenatal visits (%,n)**								
First trimester	38.9 (1045)	43.6 (2718)	0.001	76.1 (2276)	67.6 (4481)	0.001	95.6	54.7
Second trimester	42.7 (1147)	40.7 (2531)		20.5 (613)	27.3 (1810)		-52.0	-32.9
Third trimester	18.4 (495)	15.7 (971)		3.4(102)	5.1 (338)		-81.5	-67.5
**Place of prenatal visits(%,n)**								
Home/Village clinic	17.8 (478)	13.6 (845)	0.001	6.3 (189)	6.5 (431)	0.513	-64.6	-52.2
Township hospital	39.6 (1064)	52.2 (3247)		62.0 (1854)	60.9 (4037)		24.9	14.7
County health facilities	34.6 (930)	28.6 (1779)		30.7 (918)	31.8 (2108)		-	-
others	8.1 (215)	5.6 (349)		1.0 (30)	0.8 (53)		-	-
**Place of delivery (%,n)**								
Home/Village clinic	67.6 (2199)	40.3 (2896)	0.001	11.5 (350)	8.7 (586)	0.001	-83.0	-78.4
Township hospital	14.7 (478)	31.5 (2209)		47.5 (1445)	49.5 (3335)		180.7	62.6
County health facilities	16.4 (533)	24.1 (1690)		39.8 (1211)	40.9 (2755)		-	-
others	1.3 (43)	3.1 (218)		1.2 (37)	0.9 (61)		-	-
**Was mobilized to hospital delivery (%,n)**	62.7 (2040)	52.1 (3654)	0.001	94.5 (2876)	88.9 (5989)	0.001	50.7	70.6
**Maternal mortality rate (1/100,000, n)**	91.8 (37)	90.2 (76)	0.001	59.7 (22)	77.7 (71)	0.001	-34.9	-14.0

Two-sided P values for categorical variables were calculated using Pearson's χ^2 ^test, and MMR were calculated using two-sample test of Poisson distribution.

Table 3 presents the results of intervention effects from D-in-D estimation with four outcome variables adjusting for some socio-demographic characteristics. It is clear that the intervention of this program had some impacts on the utilization of prenatal care. It had significantly improved prenatal visits rate by 5.2%, first prenatal visit in first trimester rate by 12.0% and hospital delivery rate by 22.5%, respectively. And there is no effects on the rate of mobilizing pregnant women deliver in hospital compared with that of the control group.

**Table 3:** Intervention effects on utilization of prenatal health care: difference in difference (D-in-D) estimation

Outcome variable	No covariate			With covariates		
	
	D-in-D estimator (SE)	***R***^**2**^	*P*	D-in-D estimator (SE)	***R***^**2**^	*P*
Had a prenatal visit	0.058 (0.008)	0.054	0.001	0.052 (0.008)	0.085	0.001
First prenatal visit in first trimester	0.134 (0.015)	0.083	0.001	0.120 (0.015)	0.103	0.001
Had a hospital delivery	0.231 (0.012)	0.210	0.001	0.225 (0.013)	0.244	0.001
Was mobilized to hospital delivery	-0.050(0.016)	0.181	0.001	-0.037 (0.021)	0.187	0.073

The difference in difference estimator was estimated using linear regression model adjusting for covariates including women's age, women's ethnicity, parity, the distance between village and township, and average income of village per year.

## Discussion

The data of this study was collected from a survey conducted in the most underdeveloped areas in China. Most of those areas are mountainous, where the healthcare services are poor. According to a survey conducted in rural areas among five provinces of western China in 1999, the average proportion of prenatal visit rate was 73.0%, the average proportion of hospital delivery rate was 28.3% and the average proportion of prenatal visits in the first trimester was 11.5%. However, great differences in maternal care exist among these provinces. The situation is even worse in some rural areas [7].

As can be seen from the baseline survey in the intervention group in 2001, 82.6% of pregnant women had prenatal visits. However, only 38.9% of those women had their first prenatal visits in the first trimester, 74.2% of pregnant women had their prenatal visits in township hospitals or higher level hospitals, and more than half of the pregnant women gave birth at home. These indicators showed a low utilization of prenatal healthcare service and did not meet the national targets [8,9]. At the same time, the MMR was 91.8 per 100,000 live births, which was nearly two times as high as that of the whole nation, three times as high as that of the urban population and was higher than rural population [4]. Poor health performance is always linked to bad conditions, the proportion of pregnant women whose average annual incomes is less than 2,000 RMB is 98.4% and average education is 5.1 years.

The key objective of "Safe Motherhood" program was to reduce the MMR in western rural areas by improving the hospital delivery in township and eliminating the home delivery or village clinic delivery. The strategies of the program were fulfilled by improving the basic emergency obstetric care and strengthening the maternal health-service quality. Thus, strong government support was consistently provided throughout the program by county-township-village three-level health care network, which include being responsible for supervising those who carried out the project (e.g., specialists at the province level to supervise and support the township doctors), limiting the cost of hospital delivery and supplying subsides to poor pregnant women. The community-based health education effectively changed the wrong ideas about health and traditional customs predominate in rural women, such as a preference for home delivery. Moreover, village doctors rather than a midwife could give maternal health suggestion to pregnant women, and encourage them to delivery in township hospital. It avoided the maternal risks effectively. After a four-year intervention, the strategies of intervention looked effective. The prenatal visits rate was increased to 98.3%, 76.1% of those women had their first prenatal visit in the first trimester, 87.3% of pregnant women delivered in township or higher lever hospitals and MMR was reduced to 59.7 per 100,000 live births. The indicators of prenatal care utilization were better than that of the whole nation in 2005 respectively, but the MMR was still higher than that of rural population (53.9 per 100,000 live births) [4]. It can be concluded from the results that the intervention significantly increased the proportion of prenatal visits by 5.2%; first prenatal visit in the first trimester by 12.0% and 92.7% of pregnant women had their prenatal visits in township or higher lever hospitals. The rate of hospital delivery was increased by 22.5%. No effect was found on rate of being mobilized to hospital delivery compared with that of the control group, this could be due to the implementation of some policies. Chinese government has issued policies to improve maternal health care and reduce MMR in rural areas. These policies parallel the improvements in maternal health care service and status in China. The government encouraged women to give birth in hospital by varied propaganda such as TV, broadcast and posters. The pregnant woman not only was mobilized to give birth in hospital by village doctors, but also by her relatives, neighbors and friends. This could improve the rate of being mobilized to hospital delivery in control areas.

Since the "Safe Motherhood Initiative" was launched in 1987, available data have shown that the MMR has remained appalling in developing country [10-12]. It was expected that effective prenatal health care would reduce the MMR. However, based on the present study, factors which are responsible for low utilization of prenatal health include a variety of interplaying social, economic and health system factors, which operate at various levels-the household, community, the health institutions and the larger social and political environment [1,12-18]. These factors interact in different ways to determine use of health care. Maternal health care needs to be a linked system operating at different levels. For example, in Enugu, southwestern Nigeria even where maternity care services are reasonably available, the percentage of delivery in health facilities was still low [10]. In Pakistan, inappropriate strategies, inefficiency, mismanagement of finance, and lack of coordination in both public and sectors have resulted in slow progress in prenatal health care [11].

The successful experiences of the intervention measures in China suggested that strong government policy and financial support are very crucial to a community- based intervention program. On the other hand, the comprehensive intervention strategies should include technical support, manpower training, health education, community mobilization and participation. None of the above measurement seems dominate [19].

The "Safe Motherhood" program has improved utilization of maternal care and reduced MMR in target counties. But it is obvious that the great regional disparity has remained. In 2005, the hospital delivery rate was 73.6% in Xinjiang and 59.4% in Guizhou, respectively. The MMR was 147.5 per 100,000 per live births in Xinjiang and 103.5 per 100,000 live births in Qinghai, respectively. Furthermore, those selected counties in this program got great financial input and improved health care. However, what about the sustainability of health care after the program? Therefore, it is necessary to explore a practical, sustainable development and low-cost maternal and children's health model for western rural areas.

There are a number of limitations of this study. First, the counties included in the survey for the study were purposely selected based on socioeconomic and maternal health care conditions which may not be the representative of the whole western rural areas of China. Second, no quality indicators are included in the present data, making it impossible for further analyses of the intervention effects. There was also recall bias which is likely to occur because the subjects were interviewed about events occurred several years ago. At last, this is not a randomized controlled study and therefore the observed differences in the outcome variables are subject to many unobserved confounding factors.

## Conclusions

The "Safe Motherhood" program in western China seems have positive impacts on prenatal care and hospital delivery in those ten rural areas. The intervention strategies could be expanded in other rural areas. The Chinese government should assign more resources to help western poor areas build stronger capacity to carry out prenatal health care system.

## Competing interests

The authors declare that they have no competing interests.

## Authors' contributions

All authors read and approved the final manuscript. X. L. designed the prescription study, collected the data, conducted the data analysis and prepared the manuscript; H. Y. contributed to the design and analysis of the study and the preparation of the manuscript; and D. W. assisted with the data analysis and reviewed the manuscript.

## Pre-publication history

The pre-publication history for this paper can be accessed here:

http://www.biomedcentral.com/1471-2458/10/566/prepub
